# Bone Morphogenetic Proteins (BMPs), Extracellular Matrix Metalloproteinases Inducer (EMMPRIN), and Macrophage Migration Inhibitory Factor (MIF): Usefulness in the Assessment of Tubular Dysfunction Related to Chronic Kidney Disease (CKD)

**DOI:** 10.3390/jcm10214893

**Published:** 2021-10-23

**Authors:** Kinga Musiał, Danuta Zwolińska

**Affiliations:** Department of Pediatric Nephrology, Wrocław Medical University, Borowska 213, 50-556 Wrocław, Poland; danuta.zwolinska@umw.edu.pl

**Keywords:** bone morphogenetic protein (BMP)-2, bone morphogenetic protein (BMP)-6, extracellular matrix metalloproteinases inducer (EMMPRIN), macrophage migration inhibitory factor (MIF), tubular functional reserve, tubular damage

## Abstract

Bone morphogenetic proteins (BMP), extracellular matrix metalloproteinases inducer (EMMPRIN), and macrophage migration inhibitory factor (MIF) are known to be closely connected to renal tubule damage by experimental data; however, this has not been analyzed in children with chronic kidney disease (CKD). The aim of this study was to determine their usefulness in the assessment of CKD-related tubular dysfunction. The study group consisted of 61 children with CKD stages 1–5 and 23 controls. The serum and urine concentrations of BMP-2, BMP-6, EMMPRIN, and MIF were assessed by ELISA and their fractional excretion (FE) was calculated. The serum and urine concentrations of BMP-2, BMP-6, EMMPRIN, and MIF were significantly elevated in children with CKD vs. controls. The FE of BMP-2, FE BMP-6, and EMMPRIN increased significantly in CKD stages 1–2, but exceeded 1% in CKD stages 3–5. FE MIF became higher than in controls no sooner than in CKD 3–5, but remained below 1%. The FE values for BMP-2, BMP-6, and EMMPRIN of <1% may result from the tubular adaptive mechanisms, whereas those surpassing 1% suggest irreversible tubular damage. The analysis of serum/urinary concentrations and fractional excretion of examined parameters may allow the assessment of CKD-related tubular dysfunction.

## 1. Introduction

Tubular damage is an early marker of kidney injury and a major determinant of renal outcome [1]. The regenerative potential of renal tubules is clearly seen in the course of acute tubular necrosis, when the increased fractional excretion of sodium results from the timely dysfunction of proximal tubules and allows sequential control until its normalization preceding recovery. The working hypothesis is that similar follow-up of tubular reabsorption capacity is available in the course of chronic kidney disease (CKD) and that it is possible to define a breakthrough point at which reversal of tubular dysfunction is no longer possible [2]. The breakthrough is usually preceded by compensatory mechanisms aimed at maintaining the status quo. One of the nephrological examples is glomerular functional reserve, which defines the ability of glomeruli to increase filtration under unfavorable conditions. Such a situation occurs in patients with CKD stage 1, who demonstrate normal or even increased values of estimated glomerular filtration rate (eGFR), even though the features of kidney damage, such as decreased nephron mass, are already present. Similarly, tubular functional reserve should identify the ability of tubules to respond to the increasing urinary net content of various molecules during the course of CKD. Previous attempts have focused on the tubular secretory response to creatinine load as a tool to detect the subclinical condition of reduced nephron mass [3,4]. A furosemide stress test gave way to a more clinical approach [5]. However, no standardized test to assess tubular reserve has yet been developed.

From a clinical perspective, it is essential to determine a turning point at which tubular reabsorption is no longer able to mitigate the increasing net content of various substances in the urine. To evaluate the process, both specific markers and an effective method of tubular function assessment are needed. Classical markers of tubular damage, such as kidney injury molecule (KIM)-1 or α1microglobulin, were promising in the assessment of subclinical kidney injury and the prediction of renal function deterioration in patients with normal kidney function, but failed in patients with chronic kidney injury [6,7]. Therefore, when screening for suitable parameters, molecules with regenerative potential towards renal tubules may serve as candidate markers of their adaptive abilities.

Bone morphogenetic proteins (BMPs) are the members of the transforming growth factor (TGF)-β superfamily, which is responsible for cell proliferation and regeneration [8]. Animal studies have demonstrated close relations between the decreased expression of BMP-6 and damage to renal tubular cells [9]. BMP-6 null mice present more extensive tubular epithelial necrosis than their wild-type littermates [9]. BMP-2 also appears to play an important role in the regeneration of tubular cells; increased BMP-2 expression in the course of acute kidney injury was shown to induce the myofibroblastic transition in renal progenitor cells [10].

Other potential markers of tubular function are the molecules localized within the tubular structures, which will react directly to the in situ injury. Extracellular matrix metalloproteinases inducer (EMMPRIN) is expressed on the basolateral side of tubular epithelial cells. When these cells are injured in the course of AKI, EMMPRIN expression decreases as it is excreted in the urine [11]. Such a decrease has been also noted in advanced stages of fibrosis compared with the early phase [12]. Our previous studies in children with advanced CKD revealed increased serum and urinary concentrations of EMMPRIN [13,14]. Tubular macrophage migration inhibitory factor (MIF) has been characterized as an endogenous renoprotective factor, attenuating the progression of kidney damage [15]. Its activity is closely connected with the M1 subpopulation of macrophages, triggering inflammatory and regenerative responses, which lead to extracellular matrix deposition [16].

To assess the dynamic changes in tubular function, we decided to evaluate the fractional excretion of BMPs, EMMRIN, and MIF, taking into account the difference in proportion between their serum and urine pools in relation to corresponding creatinine concentrations. This compilation enables the interpretation of the behavior of a selected molecule under different CKD conditions, enriched by the context of glomerular (serum creatinine) and tubular (urinary creatinine) function. The abovementioned parameters have not previously been analyzed as markers of tubular damage in children with CKD. Moreover, nothing is known about the impact of tubular functional reserve, expressed as the values of urinary fractional excretion of the selected parameters, in CKD.

## 2. Aim of Study

Therefore, the aim of this study was to analyze the ability of renal tubules to adapt to changeable conditions, namely decreased eGFR, by assessing serum and urine concentrations of BMP-2, BMP-6, EMMPRIN, and MIF in the consecutive stages of CKD. We also assessed the fractional excretion (FE) values of BMP-2, BMP-6, EMMPRIN, and MIF in children with CKD and in controls, in order to evaluate their potential usefulness as markers of tubular dysfunction.

## 3. Methods

### 3.1. Study Design and Sampling

This was a single-center cross-sectional study concerning 61 children with CKD and 23 controls. Blood samples were drawn from peripheral veins after an overnight fast. Samples were clotted for 30 min and centrifuged for 10 min. Then serum was stored at −20 °C until being assayed. Urine from the first morning sample was collected aseptically, centrifuged for 10 min, and then stored at −20 °C until being assayed.

### 3.2. Assay Characteristics

The serum and urine concentrations of BMP-2, BMP-6, EMMPRIN, and MIF were evaluated by commercially available ELISA kits: BMP-2 (Cloud-Clone Corp., Houston, TX, USA), reagent kit SEA013Hu; BMP-6 (Cloud-Clone Corp., Houston, TX, USA), reagent kit SEA646Hu; EMMPRIN (R&D Systems, Minneapolis, MN, USA), reagent kit DEMP00; MIF (R&D Systems, Minneapolis, MN, USA), reagent kit PDMF00B. Standards, serum and urine samples were transferred to 96-well microplates pre-coated with recombinant antibodies to human BMP-2, BMP-6, EMMPRIN, and MIF. Measurements were performed in according to the manufacturer’s instructions, and results were calculated by reference to standard curves.

Serum and urine chemical parameters were measured using automated routine diagnostic tests. The serum and urine creatinine were assessed using the Creatinine OSR61204 reagent (Beckman Coulter, Aurora, OH, USA) on the Beckman Coulter AU2700 analyzer (Beckman Coulter, Aurora, OH, USA). Estimated glomerular filtration rate (eGFR) was calculated using the Schwartz formula [17]. All urinary concentrations of evaluated parameters were normalized to urinary creatinine values.

The fractional excretion (FE) of a parameter with urine was calculated according to the formula: FE [%] = ((urine parameter concentration) × (serum creatinine concentration)) ÷ ((serum parameter concentration) × (urine creatinine concentration)) × 100.

### 3.3. Statistical Analysis

The results were expressed as median values and interquartile ranges. The null hypothesis of normality of distribution was rejected by the Shapiro–Wilk test. Thus, the comparisons between variables were evaluated by using nonparametric tests (Kruskal–Wallis, Mann–Whitney U). Relations between parameters were defined by Spearman’s correlation coefficient R. Statistical analysis was performed using the package Statistica ver. 13.3 (StatSoft Inc., Tulsa, OK, USA). A *p* value of <0.05 was considered significant.

## 4. Results

### 4.1. Patient Characteristics

Eighty-four patients were divided into three groups. The first group (CKD I) consisted of 20 children with CKD stages 1–2, the second group (CKD II) contained 41 patients with CKD stages 3–5, and the control group consisted of 23 children with monosymptomatic nocturnal enuresis and normal kidney function. The basic clinical data are presented in Table 1.

The major causative factors for CKD were congenital anomalies of the kidney and urinary tract (CAKUT) (45 children), including reflux nephropathy (19 cases), obstructive uropathy (14 patients), and hypo/dysplastic kidneys (12). Other underlying diseases were: chronic glomerulonephritis (10), polycystic kidney disease (4), and hemolytic uremic syndrome (2). CAKUT was the only cause of CKD in CKD I and the dominant cause in CKD II (Table 1).

Patients did not show clinical evidence of infection and did not smoke or take antibiotics or statins. They were also free of such co-morbidities as diabetes, malignancies, connective tissue diseases, cardiovascular disease, peripheral vascular disease, or obesity. Thirty-five children from the CKD group were normotensive based on the criteria of the European Society of Hypertension in children and adolescents [18]. Sixteen patients had clinically well-controlled blood pressure, using ACE inhibitors (8 children), calcium channel blockers (6 patients), and β-blockers (2 children); 10 patients required combined therapy. All patients with CKD stages 3–5 were supplemented with phosphate binders and vitamin D metabolites.

### 4.2. Serum and Urine Concentrations of BMP-2, BMP-6, EMMPRIN, and MIF

The serum concentrations of all examined parameters in CKD patients were significantly higher than in controls, irrespective of the stage of the disease (Table 2). Serum BMP-2 and BMP-6 levels reached their maximum in early stages of CKD (CKD I) and then decreased significantly throughout advanced CKD (CKD II), although remained elevated vs. controls. In contrast, serum EMMPRIN and MIF values continued to increase as CKD progressed (Table 2).

The urine concentrations of BMP-2, BMP-6, and EMMPRIN were higher in children with CKD compared with the controls and gradually increased with CKD progression (Table 3). The MIF values increased in CKD I vs. the control and then reached a plateau phase between mild (CKD I) and advanced (CKD II) CKD (Table 3). Urine creatinine concentrations were elevated in patients with CKD I in comparison with the controls, but in the CKD II group they were lower than in the CKD I and control group (Table 1).

### 4.3. Fractional Excretion of BMP-2, BMP-6, EMMPRIN, and MIF

The fractional excretion (FE) values of BMP-2, BMP-6, and EMMPRIN were significantly elevated in all children with CKD when compared with the control group, and increased with the progression of CKD (Table 4). However, FE BMP-2, FE BMP-6, and FE EMMPRIN in mild CKD remained below 1% and exceeded this threshold no sooner than in the CKD II group. The FE values of BMP-2, BMP-6, EMMPRIN, and MIF in the control group were all below 1%. FE MIF values remained unchanged in the CKD I group vs. the control group, and then increased significantly in the CKD II group, but did not exceed 1% (Table 4).

### 4.4. Correlations

The BMP-2, BMP-6, EMMPRIN, and MIF serum concentrations were significantly correlated with each other, with eGFR, and with the corresponding urine values (Table 5).

The urine concentrations of all the analyzed parameters were also correlated with each other, and the strength of these correlations was more significant than for the serum values (−0.54 ≤ R ≤ −0.49; *p* < 0.0001). The urine concentrations of BMP-2, BMP-6, EMMPRIN, and MIF were negatively correlated with eGFR (−0.59 ≤ R ≤ −0.55; *p* < 0.0001).

## 5. Discussion

Our investigation revealed changes in the serum and urine concentrations of BMP-2, BMP-6, EMMPRIN, and MIF, as well as adaptive changes in the FE values, in children with CKD when compared with the controls. The kinetics of these changes were dependent upon the analyzed molecule.

### 5.1. Bone Morphogenetic Proteins

The serum concentrations of BMP-2 and BMP-6 followed similar patterns to each other, but were different to those of other examined molecules. An early rise in CKD stages 1–2 was followed by a statistically significant, although not large, decrease in CKD stages 3–5. However, the concentrations remained higher than those in the controls. The serum values of BMPs were positively correlated with eGFR, which could explain the decrease in their concentration as CKD progressed. The progressively increasing urinary concentrations of BMP-2 and BMP-6 were strongly dependent on the eGFR values. Moreover, BMPs are low-molecular-weight proteins, so they can be freely filtered into urine. Thus, the decrease in serum BMPs during the late stages of CKD can be partially explained by the excretion of molecules in the urine. Meanwhile, the FE BMP-2 and BMP-6 values, which increased with the progression of CKD, but did not exceed the 1% threshold in CKD stages 1–2, could act as surrogate markers for the enhanced tubular activity. Experimental studies suggest that the change in creatinine tubular secretion, triggered, e.g., by protein meal, may serve as a marker of tubular functional reserve [19]. Indeed, such overactivity was seen in our study, where children with CKD I demonstrated increased creatinine urine concentrations compared with the controls. This may be indirect proof of aggravated tubular activity. Such mobilization of the tubular functional reserve would aid the reabsorption of excessive urinary BMPs in order to prevent the loss of filtered proteins. Crossing the borderline of 1% in advanced CKD indicates that the renal tubules are unable to cope with the urinary protein overload. Indeed, this exhaustion of renal tubule function was clearly shown by the urine creatinine values, decreasing to the level below that of the control group owing to the impaired secretory abilities of the tubules. Thus, the simultaneous analysis of serum/urine/fractional excretion values broadens the horizon of dynamic adaptive changes that the tubules may undergo during CKD progression.

### 5.2. EMMPRIN

The decrease in EMMPRIN expression has been demonstrated in human kidney allografts, along with the progression of fibrosis and tubular atrophy [12]. Increased plasma and urinary EMMPRIN levels have been reported in patients with tubular atrophy/interstitial fibrosis in the course of IgA nephropathy and diabetic kidney disease [20]. Moreover, the strong correlation of both plasma and urinary EMMPRIN with eGFR has been noted [20]. Therefore, EMMPRIN has been identified as a marker that can accurately reflect disease activity and tubular damage. In our study, EMMPRIN serum and urinary concentrations, as well as FE values, also increased systematically and significantly with CKD progression. Likewise, both serum and urine EMMPRIN values were correlated negatively with eGFR. Thus, the gradual increase in serum can be explained by both the accumulation and overproduction during disease progression, whereas the increase in EMMPRIN in urine can be explained by the cumulative effects of molecule leakage and release by damaged tubular cells into urine. Consequently, this would justify the rise in FE values from the earliest stages of CKD and the surpassing of the 1% borderline, indicating the adaptive ability of tubular function in early CKD, as well as the irreversible tubular damage in advanced CKD and an inability to acquire the increased net content of EMMPRIN in the urine.

### 5.3. MIF

MIF is an anti-inflammatory cytokine that inhibits migration of macrophages and triggers their adhesion and accumulation at the sites of inflammation and phagocytosis. Increased serum concentrations of MIF have been reported in adults with uremic cardiomyopathy [21]. Therefore, the MIF systemic pool appears to represent destructive mechanisms in the course of CKD. The gradual increase in serum MIF in our patients was concordant with previous results and could indicate the accumulation of the molecule. An additional argument for this explanation is the negative correlation with eGFR. In contrast, owing to its low molecular weight, MIF could be freely filtered through a glomerular filtration barrier. Thus, the MIF overproduction in the systemic pool due to the chronic inflammatory process, which is characteristic for CKD, was also likely. Moreover, the increase in serum MIF concentration was noticeable, despite the undisturbed elimination of the molecule in urine; thus, the cumulative effect of accumulation and overproduction was the most likely explanation.

The increased MIF in urine in the early stages of CKD was the most notable among all parameters, whereas urinary concentrations in advanced CKD remained stable despite the increasing serum values, unlike other markers. MIF activity in the kidney is strongly dependent on the presence of M1 macrophages, which are predominant in the early period of injury but give way to type M2 in advanced fibrosis [16]. Therefore, the abrupt early MIF increase in urine could indicate the cumulative effects of molecule leakage and its protective overactivity in situ. Meanwhile, the stable urinary values of MIF in CKD stages 3–5 vs. stages 1–2, as well as their negative correlation with eGFR, suggest a suppression of pro-inflammatory activity in response to the progression of fibrosis, which is characteristic of the advanced stages of CKD. They may also be an indirect marker of the switch from the pro-inflammatory M1 macrophage phenotype to M2. Tubular MIF also has been known for its regenerative potential during the course of AKI [22,23]. Therefore, the increase in urinary MIF may serve as proof of its regenerative activity in the course of early CKD, whereas the later plateau phase toward advanced CKD may illustrate that this activity is exhausted and the transition of active inflammatory process creates irreversible damage. Consequently, owing to the various stimuli that influence MIF concentrations, FE MIF values only increased to higher than the control group in CKD stages 3–5 and they did not exceed 1% in any of the analyzed groups.

### 5.4. The Role of Fractional Excretion

Collectively, the complex analysis of serum, urine, and fractional excretion values of BMPs and EMMPRIN enabled the differentiation between the mobilization of tubular functional reserve in the face of increased parameter urine load and the subsequent tubular damage during the progression of CKD. The value of the obtained results was strengthened by the fact that the critical period of tubular adaptive activity was analyzed in a group of children with CAKUT as a major reason for CKD development. This homogeneity allowed the exclusion of any potential glomerular interference from the background of dysfunction. The analyzed group became more heterogenous in advanced stages of CKD, when tubular damage is common to all underlying diseases leading to CKD.

Fractional excretion has been shown to be a useful tool in the assessment of CKD-related tubular dysfunction, provided that free filtration of the molecule was the major mechanism of its elimination in urine. Given that none of the FE values in the control group exceeded 1%, we could assume that none of the examined molecules were actively secreted by the tubules. However, when additional mechanisms were considered to contribute to the final serum and urine concentrations, such as inflammation or macrophage transition/fibrosis in the case of MIF, this analysis yielded inconclusive results.

We must also acknowledge the limitations of our study. First, the heterogeneity of factors influencing serum and urine concentrations of BMPs, EMMPRIN, and MIF, as well as a cross-sectional design, could create a bias and make the interpretation of their increased/decreased/stable concentrations a challenge. The narrow range in FE variation may also increase difficulties in the proper interpretation of obtained results. However, our main goal was to evaluate the proportion between the serum and urinary pools and identify a common pattern of tubular adaptive mechanisms, rather than analyze the selected molecules separately. Second, our hypothesis is new and this is the first attempt to use FE as a tool to assess tubular activity in chronic conditions, so our results cannot be compared with any previous data from experimental or physiology studies. We are also aware of the small number of patients, which limits the power of our conclusions and necessitates continued study of a larger group of patients.

## 6. Conclusions

The FE values of BMP-2, BMP-6, and EMMPRIN, which were below 1% in CKD stages 1–2, may result from adaptive tubular mechanisms, whereas FE values surpassing the 1% threshold in CKD stages 3–5 suggest irreversible tubular damage.

The complex analysis of serum/urinary concentrations and FE values of multiple parameters may demonstrate the features of CKD-related tubular dysfunction.

## Figures and Tables

**Table 1:** Basic clinical characteristics of the patients.

Parameter	Median (Lower–Upper Quartile)		
	Control Group (*n* = 23)	CKD I (*n* = 20)	CKD II (*n* = 41)
Age (years)	10.5 (5.0–16.5)	9.5 (4.0–12.5)	11.0 (4.5–16.5)
Gender	13 girls, 10 boys	5 girls, 15 boys	17 girls, 24 boys
Primary cause of CKD	-	CAKUT/GN/other	CAKUT/GN/other
		20/0/0	25/10/6
Serum creatinine [mg/dL]	0.6 (0.5–0.7)	1.1 (1.0–1.2) ^a^	1.9 (1.3–3.7) ^b^
Urine creatinine [mg/dL]	114 (100–126)	131 (118–140) ^a^	76 (60–82) ^b^
Proteinuria [g/L]	0.01 (0.0–0.1)	0.02 (0.02–0.2)	0.4 (0.03–0.6) ^b^
eGFR [mL/min/1.73 m^2^]	97.1 (92.3–115.0)	79.5 (65.7–97.4) ^a^	26.2 (17.3–41.5) ^b^

Mann–Whitney U test: ^a^ *p* < 0.001 CKD I vs. control gr.; ^b^ *p* < 0.001 CKD II vs. CKD I; CAKUT, congenital anomalies of the kidney and urinary tract; GN, glomerulonephritis.

**Table 2:** Serum concentrations of examined parameters in children with CKD and in the control group.

Parameters in Serum	Median Value (Lower–Upper Quartile)		
	Control Group (*n* = 23)	CKD I (*n* = 20)	CKD II (*n* = 41)
sBMP-2 [pg/mL]	523.8 (513.8–538.9)	1574.1 (1540.1–1645.1) ^a^	1527.6 (1495.9–1570.5) ^b^
sBMP-6 [ng/mL]	33.3 (31.8–34.7)	96.6 (95.9–97.7) ^a^	92.5 (90.6–96.3) ^b^
sEMMPRIN [pg/mL]	871.9 (854.9–906.1)	1114.4 (1085.1–1137.8) ^a^	1175 (1150.6–1199.4) ^b^
sMIF [ng/mL]	20.1 (19.4–21.4)	61.9 (61.7–62.4) ^a^	66.5 (62.4–68.5) ^b^

Mann–Whitney U test: ^a^ *p* < 0.0001 CKD I vs. control gr.; ^b^ *p* < 0.001 CKD II vs. CKD I.

**Table 3:** Urine concentrations of examined parameters in children with CKD and in the control group.

Parameters in Urine	Median Value (Lower–Upper Quartile)		
	Control Group (*n* = 23)	CKD I (*n* = 20)	CKD II (*n* = 41)
uBMP-2 [pg/mg creatinine]	160.3 (146.2–198.1)	571.8 (516.7–611.7) ^a^	900.7 (797.7–1172.4) ^b^
uBMP-6 [ng/mg creatinine]	12.4 (11.4–14.7)	39.8 (37.2–44.3) ^a^	62.2 (56.5–84.4) ^b^
uEMMPRIN [pg/mg creatinine]	375 (313.3–402.4)	629.2 (572.2–690.1) ^a^	1117.9 (962.1–1361.5) ^b^
uMIF [ng/mg creatinine]	0.9 (0.8–1.1)	3.5 (3.2–3.7) ^a^	3.8 (3.4–4.6) ^b^

Mann–Whitney U test: ^a^ *p* < 0.0001 CKD I vs. control gr.; ^b^ *p* < 0.01 CKD II vs. CKD I.

**Table 4:** Fractional excretion of examined parameters in children with CKD and in the control group.

Fractional Excretion of Parameters	Median Value (Lower–Upper Quartile)		
	Control Group (*n* = 23)	CKD I (*n* = 20)	CKD II (*n* = 41)
FE BMP-2 [%]	0.21 (0.19–0.24)	0.39 (0.35–0.45) ^a^	0.74 (0.45–1.42) ^b^
FE BMP-6 [%]	0.27 (0.24–0.28)	0.45 (0.41–0.54) ^a^	0.81 (0.54–1.78) ^b^
FE EMMPRIN [%]	0.30 (0.28–0.31)	0.62 (0.57–0.72) ^a^	1.12 (0.73–2.13) ^b^
FE MIF [%]	0.03 (0.02–0.04)	0.03 (0.02–0.04) ^a^	0.06 (0.04–0.14) ^b^

Mann–Whitney U test: ^a^ *p* < 0.0001 CKD I vs. the control group; ^b^ *p* < 0.00001 CKD II vs. CKD I.

**Table 5:** Selected correlations between serum (s) and urine (u) parameters in children with CKD assessed by Spearman’s correlation coefficient (R).

Examined Parameters	sBMP-2	sBMP-6	sEMMPRIN	sMIF	eGFR	Urine Corresponding Values
sBMP-2	-	R = 0.49, *p* < 0.0001	R = −0.35, *p* < 0.01	R = −0.29, *p* < 0.01	R = 0.34, *p* < 0.01	uBMP-2 R = 0.02, *p* = 0.8
sBMP-6	R = 0.49, *p* < 0.0001	-	R = −0.61, *p* < 0.00001	R = −0.66, *p* < 0.00001	R = 0.63, *p* < 0.00001	uBMP-6 R = −0.55, *p* < 0.0001
sEMMPRIN	R = −0.35, *p* < 0.01	R = −0.61, *p* < 0.00001	-	R = 0.57, *p* < 0.00001	R = −0.62, *p* < 0.00001	uEMMPRIN R = 0.48, *p* < 0.0001
sMIF	R = −0.29, *p* < 0.01	R = −0.66, *p* < 0.00001	R = 0.57, *p* < 0.00001	-	R = −0.53, *p* < 0.0001	uMIF R = 0.78, *p* < 0.000001

## Data Availability

The datasets generated and analyzed during the current study are available from the corresponding author on reasonable request.
